# Supplementary figures and images for: One Health Disparities and Blastocystis infection among smallholder farmers in northeastern Madagascar
Source: PLOS Glob Public Health. 2025 Oct 13;5(10):e0005189. doi: 10.1371/journal.pgph.0005189 (PMC12517528; doi:10.1371/journal.pgph.0005189)

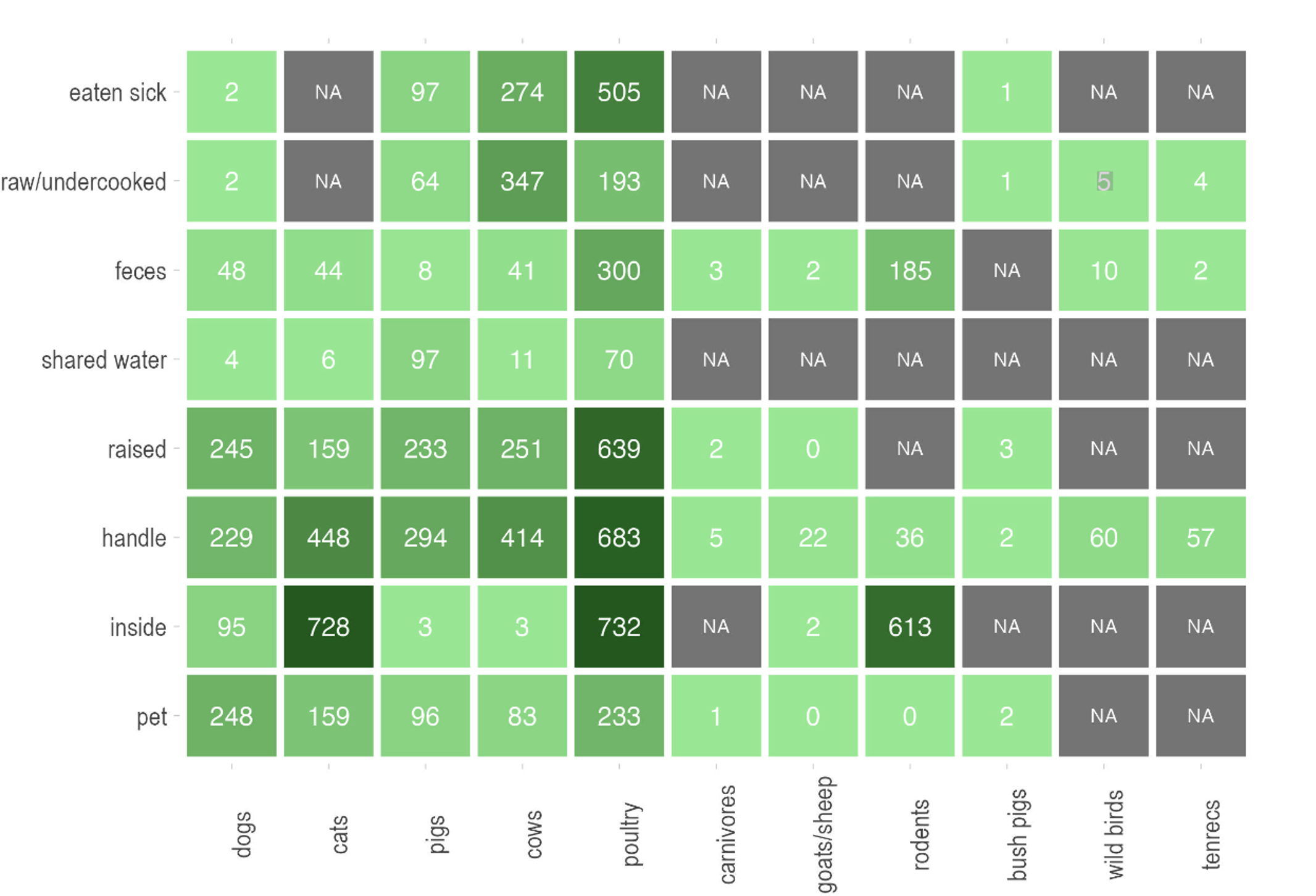

Supplement: S1 Fig — The total reported interactions with 11 animal species (dogs, cats, pigs, cows, poultry, carnivores, goats/sheep, rodents, bush pigs, wild birds, and tenrecs) and the total number of 8 interaction types (e.g., consumed when sick, consumed raw/undercooked, feces near food, shared water, raised, handled, kept inside dwelling, and kept as pets). “NA” indicates no reported interaction for a specific animal species. (TIF) [file pgph.0005189.s001.tif]

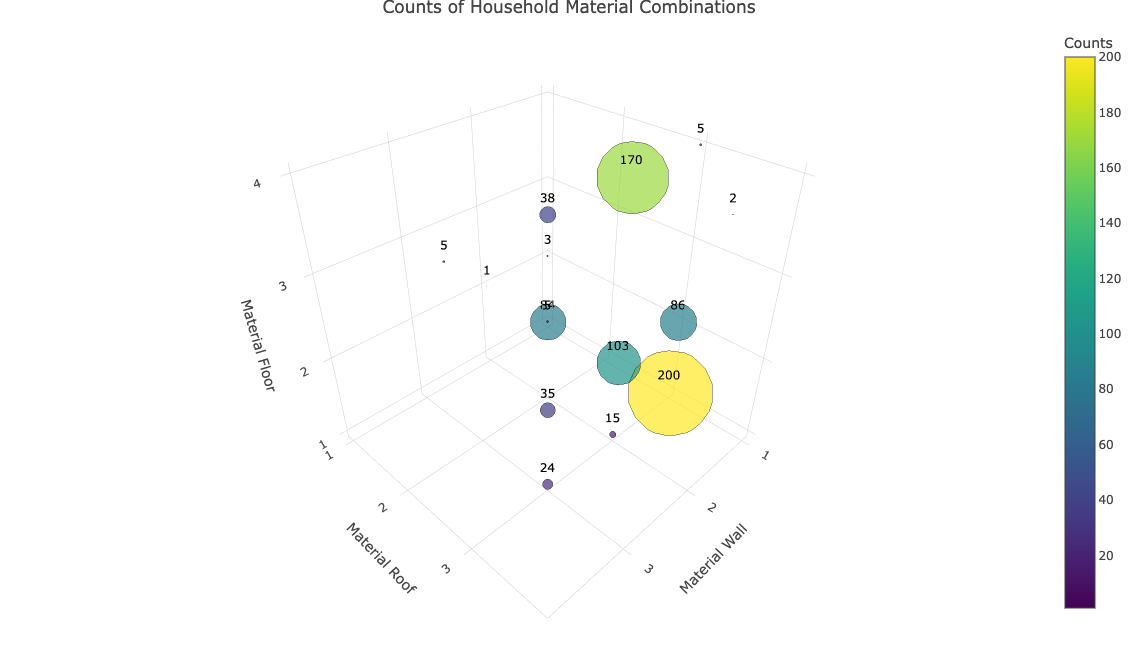

Supplement: S2 Fig — Reported materials used for household outer walls, roofs, and floors were assessed through three questions with identical response options: thatch, compacted earth, burnt brick, metal sheets (iron, aluminum), mud, mud brick, concrete/cement, bamboo, wood planks, travelers palm, and raffia palm. Each material was ranked numerically from 1 to 4 based on local availability and cost. The scores from the three categories were summed to calculate a housing material index score, ranging from 3 to 12. Circle size and color represent the count/frequency of reported housing material scores. (TIF) [file pgph.0005189.s002.tif]
