# Supplementary material for: One Health Disparities and Blastocystis infection among smallholder farmers in northeastern Madagascar
Source: PLOS Glob Public Health. 2025 Oct 13;5(10):e0005189. doi: 10.1371/journal.pgph.0005189 (PMC12517528; doi:10.1371/journal.pgph.0005189)
Supplement: Attachment — Submitted filename: Responses.Reviewers.Final.pdf [file pgph.0005189.s007.pdf]

Response to Reviewers
Manuscript Title: One Health Disparities and Blastocystis infection among smallholder farmers in northeastern Madagascar
Manuscript ID: PGPH-D-25-00246
Corresponding Author: Alma Solis, M.S., Ph.D., Duke University
June 30th, 2025

Dear Editors,

We thank you for the opportunity to revise our manuscript and for the insightful and constructive comments. We have carefully addressed all points raised and provide our detailed responses below. We believe these changes have improved the clarity and impact of the manuscript.

Below are the comments we addressed:

PLOS Global Public Health Reviewer Comments:

Comment 1: Please can you add how the participants were selected; was it random or purposive?

RESPONSE: We added the following text to explain the recruitment strategy
Recruitment occurred between October 3rd 2019 and August 8th 2022 using snowball sampling, in which surveys included name generators to create lists of individuals to recruit for subsequent surveys (Kauffman et al. 2019)

Comment 2: Explain the wide recruitment period (2019–2023) and address any impacts on data reliability.

RESPONSE: We added a sentence in the Methods section noting that the wide recruitment period was spread out to align with different rainy and dry seasons as part of a larger study. We also adjusted the sampling period from 2019-2022 to accurately reflect our recruitment period.

Comment 3: (first hypoothesis test); it will be good to put it here within the sentences or as a sub heading

RESPONSE: We added the following text to clarify the survey design to test the first hypothesis: To test our first hypothesis related to wealth, we used two proxy numerical variables to assess wealth. To collect the two proxy wealth variables, we first asked three questions…

Comment 4: Clarify scoring range for the housing material index and which scores are high or low.

RESPONSE: We clarify that scores ranged from 3 to 12, with a higher score indicating houses were constructed using more costly material.

Comment 5: Use 'CD player' instead of 'radio/CD'.

RESPONSE: We have made this correction in the manuscript and supplemental materials.

Comment 6: Did you measure or asked questions about interaction with more than one animal or multiple animals?

RESPONSE: We added clarification that animal richness variable accounts for interactions with more than one animal.

Comment 7: Also, it is unclear from the method whether the same individuals sampled for the Blastocystis screening participated in the survey, although the presentation of the results shows so. Please, clarify in your methodology.

RESPONSE: We added to the methods section that fecal samples were collected from the same survey participants.

Comment 8: Suggest adjusting large tables to fit the page.

RESPONSE: We reformatted tables to improve readability and ensure they fit standard journal margins.

Comment 9: While this study may be clear for some on the One Health franework, it will be necessary to clearly indicate what the components of the framework were used in the study and which ones were significant in the study.

RESPONSE: We applied the One Health Disparities (OHD) framework to investigate the predictors of Blastocystis infection among rural farmers in northeast Madagascar. This framework expands on the One Health approach – i.e., the interconnectedness of human, nonhuman animal, and environmental health – by incorporating the human social environment. This integrative perspective helped disentangle the underlying social determinants of how infection patterns varied by gender, handwashing practices, and village. However, OHD was less informative for understanding the role of wealth and animal contact. The findings highlight that, among the three villages studied,

variation of behavior (gender norms and handwashing) and village, are key determinants of Blastocystis infection. Understanding these sociocultural influences is crucial for effectively reducing disease risk and providing village-level interventions.

Comment 10: Explain why Village A and Village S had higher infection odds than Village M.

RESPONSE: We added the following clarification statement.
Finally, we identified that Villages A and S had significantly higher odds of infection in comparison to Village M, which may be due to variation in housing proximity, population density, or water source (26).

Comment 11: Line 378: Please, include the in-text citation for the appropriate reference
RESPONSE: In text citation was added to the original line 378

Comment 12: Line 419 - 427: Please, adjust the inconsistent text font.
RESPONSE: The text font has been adjusted to align with the rest of the manuscript

Comment 12: Ensure figures have labeled axes.

RESPONSE: We confirmed that all figures now include clearly labeled axes and legends, and updated the figure with color-blind accessibility

Comment 13: Describe updates to citations

RESPONSE: We added the following three citations:

- *Kauffman K, Werner CS, Titcomb G, Pender M, Rabezara JY, Herrera JP, et al. Comparing transmission potential networks based on social network surveys, close contacts and environmental overlap in rural Madagascar. J R Soc Interface. 2022;19(186):20210690.*

- *Coyle AH, Berrian AM, van Rooyen J, Bagnol B, Smith MH. Gender Roles and One Health Risk Factors at the Human–Livestock–Wildlife Interface, Mpumalanga Province, South Africa. EcoHealth. 2020;17(2):233-47.*

- *Shrestha S, Cortni B, and Rasolofoniaina BJR. Gender roles in natural resource use in Madagascar. Ecosystems and People. 2024;20(1):2344850.*

We also removed Barrett et al. 2025 former citation number 27 for repetition with citation number 26.

***Comment 14:** Data Sharing Policy*

RESPONSE: We updated the data accessibility statement: The data that support the findings of this study are available on request from the corresponding author. The data

are not publicly available due to privacy or ethical restrictions. Participants did not give their consent to have individual level data made public. Data requests can be made to Duke University Campus Institutional Review Board (campusirb@duke.edu) with Holly Williams (holly.williams.irb@duke.edu) being the point of contact.

Comment 15: *Request for Inclusivity Questionnaire*

RESPONSE: The questionnaire was uploaded as a supporting document.

--

We sincerely thank the reviewers and editor for their thoughtful feedback. We believe the manuscript is now significantly strengthened and look forward to the opportunity for publication.

Sincerely,

Alma Solis, M.S., Ph.D.
(on behalf of all co-authors)
Department of Evolutionary Anthropology, Duke University
alma.solis@duke.edu
